# Effects of Dietary Nitrate Supplementation on Performance during Single and Repeated Bouts of Short-Duration High-Intensity Exercise: A Systematic Review and Meta-Analysis of Randomised Controlled Trials

**DOI:** 10.3390/antiox12061194

**Published:** 2023-05-31

**Authors:** Nehal S. Alsharif, Tom Clifford, Abrar Alhebshi, Samantha N. Rowland, Stephen J. Bailey

**Affiliations:** 1 School of Sport, Exercise and Health Sciences, Loughborough University, Loughborough LE11 3TU, UK; n.alsharif@lboro.ac.uk (N.S.A.); t.clifford@lboro.ac.uk (T.C.); a.alhebshi@lboro.ac.uk (A.A.); s.rowland@lboro.ac.uk (S.N.R.); 2 Department of Clinical Nutrition, Faculty of Applied Medical Sciences, King Abdulaziz University, Jeddah 21589, Saudi Arabia; 3 Department of Clinical Nutrition, Faculty of Applied Medical Sciences, Umm Al-Qura University, Makkah 24382, Saudi Arabia

**Keywords:** nitric oxide, beetroot, exercise performance

## Abstract

Inorganic nitrate (NO_3_^−^) has emerged as a potential ergogenic aid over the last couple of decades. While recent systematic reviews and meta-analyses have suggested some small positive effects of NO_3_^−^ supplementation on performance across a range of exercise tasks, the effect of NO_3_^−^ supplementation on performance during single and repeated bouts of short-duration, high-intensity exercise is unclear. This review was conducted following PRISMA guidelines. MEDLINE and SPORTDiscus were searched from inception to January 2023. A paired analysis model for cross-over trials was incorporated to perform a random effects meta-analysis for each performance outcome and to generate standardized mean differences (SMD) between the NO_3_^−^ and placebo supplementation conditions. The systematic review and meta-analysis included 27 and 23 studies, respectively. Time to reach peak power (SMD: 0.75, *p* = 0.02), mean power output (SMD: 0.20, *p* = 0.02), and total distance covered in the Yo-Yo intermittent recovery level 1 test (SMD: 0.17, *p* < 0.0001) were all improved after NO_3_^−^ supplementation. Dietary NO_3_^−^ supplementation had small positive effects on some performance outcomes during single and repeated bouts of high-intensity exercise. Therefore, athletes competing in sports requiring single or repeated bouts of high-intensity exercise may benefit from NO_3_^−^ supplementation.

## 1. Introduction

Inorganic nitrate (NO_3_^−^) has been conventionally considered an environmental carcinogen and inert end-product of endogenous nitric oxide (NO) oxidation [1]. More recent research challenges these assertions and has revealed various potential health benefits afforded by increased dietary NO_3_^−^ intake [2]. Over the last couple of decades, dietary NO_3_^−^ supplementation has emerged as a potential nutritional strategy to improve exercise performance in healthy and moderately trained individuals [3,4]. The ergogenic effects of NO_3_^−^ supplementation have been attributed to its stepwise reduction to nitrite (NO_2_^−^) and the subsequent reduction of NO_2_^−^ to NO [2,5]. Although initially recognised for its vasodilatory properties [6], it is now appreciated that NO can positively modulate a plethora of physiological responses in skeletal muscle [7,8,9], the conflation of which is likely to underpin improved exercise performance following dietary NO_3_^−^ supplementation [5]. 

Initial studies assessing the potential efficacy of NO_3_^−^ supplementation to enhance physiological and performance responses during exercise revealed improvements in exercise economy and exercise tolerance [10,11,12]. These improvements in endurance exercise performance parameters after NO_3_^−^ supplementation were initially linked to a lower adenosine triphosphate (ATP) cost of muscle force production (improved contractile efficiency), an associated blunting in the perturbation to high-energy phosphate substrates and metabolites [13], and to a lower mitochondrial adenosine diphosphate/oxygen ratio (P/O ratio; a lower O_2_ cost of ATP resynthesis), reflecting improved mitochondrial respiratory efficiency [14]. However, the mechanisms by which NO_3_^−^ supplementation can improve exercise economy and endurance exercise performance are still to be resolved in human skeletal muscle [15,16]. 

Following on from the initial human studies, experiments conducted using murine models indicated potential fibre-type-specific effects of NO_3_^−^ supplementation on physiological responses [17]. Indeed, NO_3_^−^ supplementation was initially reported to increase calcium (Ca^2+^) handling proteins and evoked force production in type II skeletal muscle, but not slow-twitch (type I) skeletal muscle, in mice [18]. Subsequently, NO_3_^−^ supplementation increased hindlimb blood flow in exercising rats, with this additional blood flow shunted towards more fast-twitch (type II) muscle fibres [19]. The potential for enhanced efficacy of NO_3_^−^ supplementation to improve physiological and performance responses in murine type II muscle is consistent with data from human studies demonstrating enhanced pulmonary O_2_ uptake ($\dot{V}O_{2}$) and muscle deoxyhaemoglobin + deoxymyoglobin kinetics in exercise settings that evoke greater type II muscle fibre recruitment compared to exercise settings that evoke mostly type I muscle fibre recruitment [20]. Moreover, cross-sectional data have revealed that NO_3_^−^ supplementation is less likely to improve exercise economy and endurance performance as aerobic fitness increases [21], an effect that has been attributed, at least in part, to a lower % and proportional recruitment of type II muscle fibres in endurance-trained participants with a more aerobic phenotype [22]. On this basis, NO_3_^−^ supplementation may have greater potential as an ergogenic aid in exercise settings which evoke greater type II muscle fibre recruitment.

It is well documented that type II skeletal muscle fibres are recruited in an intensity-dependent manner, with greater recruitment of type II muscle fibres at higher exercise intensities [23,24,25]. In addition, the reduction of NO_2_^−^ to NO is enhanced in conditions of acidosis and hypoxia [26,27,28]. The partial pressures of O_2_ (PO_2_) and pH are lower in contracting type II than type I muscles [29,30] and progressively decline with increasing exercise intensity [31]. Therefore, high-intensity exercise, which is supramaximal with regards to the power output required to elicit $\dot{V}O_{2\max}$, and evokes significant recruitment of type II muscle fibres and declines in muscle pH and PO_2_, appears to have greater potential to elicit an ergogenic effect from NO_3_^−^ supplementation compared to continuous submaximal endurance exercise. There is also evidence to suggest that NO_3_^−^ supplementation is more effective at improving physiological and functional responses at higher, compared to lower, movement velocities [32,33]. In addition, NO_3_^−^ supplementation has been reported to increase the peak contractile velocity of, and power output generated by, contracting skeletal muscle [33,34], and to lower the time taken to achieve peak power output [35,36]. Collectively, these improvements in skeletal muscle contractile function after NO_3_^−^ supplementation would be expected to translate into enhanced single and repeated sprint performances. However, whilst there is some evidence to support an ergogenic effect of NO_3_^−^ supplementation on single and repeated bouts of short-duration large muscle mass exercise in humans (e.g., [37,38]), the existing evidence basis is equivocal (e.g., [39,40,41]). In part, these interstudy discrepancies may be attributable to disparate NO_3_^−^ supplementation and high-intensity exercise protocols, which complicates interpretation of the ergogenic potential of NO_3_^−^ supplementation for high-intensity exercise. 

Although the effects of NO_3_^−^ supplementation on performance in a variety of exercise performance tests have been systematically reviewed and have undergone meta-analyses before [42,43,44,45,46,47,48], these have not yet considered the effects of NO_3_^−^ supplementation on single and repeated bouts of short-duration large muscle mass exercise in humans. This is important to address to help improve understanding of the exercise settings in which NO_3_^−^ supplementation is ergogenic and to inform recommendations for NO_3_^−^ supplementation to improve exercise performance. Therefore, the purpose of this study was to conduct a systematic review and meta-analysis of the effects of NO_3_^−^ supplementation on single and repeated bouts of short-duration large muscle mass exercise in healthy humans. A secondary purpose was to conduct sub-analyses to evaluate the influence of the NO_3_^−^ supplementation dose and duration, participant sex, exercise type (single vs. repeated sprints), exercise duration, and plasma NO_3_^−^ and NO_2_^−^ concentrations ([NO_3_^−^] and [NO_2_^−^], respectively) to further refine understanding of the experimental conditions in which NO_3_^−^ supplementation is more likely to enhance single and repeated bouts of short-duration large muscle mass exercise. 

## 2. Materials and Methods

This systematic review and meta-analysis was reported according to Preferred Reporting items for Systematic Reviews and Meta-Analysis (PRISMA) guidelines [49]. The study protocol was registered with the Center for Open Science organisation (registration number: 10.17605/OSF.IO/JSGKM). 

### 2.1. Inclusion and Exclusion Criteria 

Three researchers (N.S.A., S.J.B., and T.C.) agreed on the inclusion and exclusion criteria. These were based on a Population, Intervention, Comparator, Outcome, Study design (PICOS) methodology (see Online Supplementary Material). Briefly, studies were included if they met the following criteria: (1) participants were healthy adults ≥16 years old; (2) they administered oral inorganic NO_3_^−^ supplements such as beetroot juice or sodium/potassium NO_3_^−^ salts and provided information about the dose, frequency, and duration of supplementation; (3) they included exercise that recruited a large muscle mass such as running, cycling, and kayaking; (4) the exercise test included ≥1 high-intensity effort (≥$\dot{V}O_{2\mathrm{peak}}$), with each effort ≤60 s; (5) they measured performance as completion time, total distance covered, maximal or mean power output, total work performed, or maximal number of repetitions. Studies were excluded if participants were <16 years old or had a chronic medical condition; NO_3_^−^ was administered with another dietary supplement; there was insufficient information about the dose, frequency, and duration of supplementation; exercise was submaximal (≤$\dot{V}O_{2\max}$) or if any single effort was ≥60 s; and if exercise was performed in hypoxic or hot conditions. 

### 2.2. Search Strategy 

We searched Medline and SPORTdiscus databases for English language papers from inception to January 2023. Our search strategy was based on our PICOS methodology and the full search terms for both databases are presented in the Online Supplementary Material. The reference lists of eligible full text articles were also searched to identify any other potential studies for inclusion. 

### 2.3. Study Selection 

**Figure 1:**
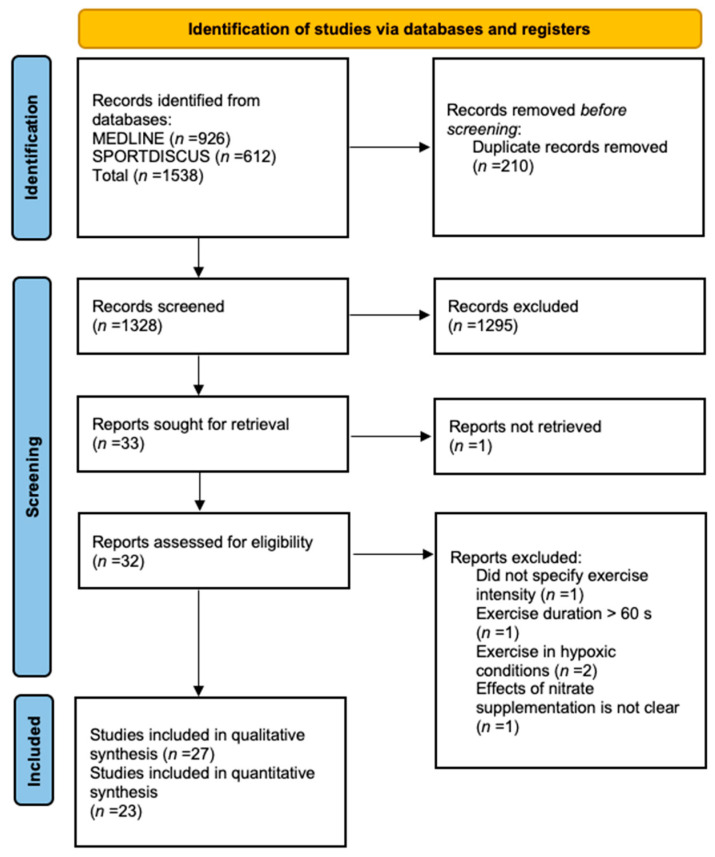
Preferred reporting items for systematic review and meta-analysis PRISMA flow diagram for study selection process. Nitrate; NO_3_^−^.

The search results were downloaded into Rayyan software, a web tool for screening abstracts [50]. After removing duplicates, two researchers (N.S.A. and S.N.R.) independently screened titles and abstracts for inclusion. Full texts of studies deemed eligible were retrieved and compared against the predefined PICOS criteria. Where there was disagreement on whether a study should be included or excluded from the systematic review and meta-analysis, this was discussed with, and resolved by, a third researcher (S.J.B.). The study selection process is summarised in Figure 1.

### 2.4. Data Extraction 

Data were extracted into a Microsoft Excel Spreadsheet by one researcher (N.S.A.) and substantiated by a second researcher (S.N.R.). The spreadsheet was designed and trialled by three authors (N.S.A., T.C., and S.J.B.) and refined prior to extraction. The following data and information were extracted: study design, sample size, participant characteristics (age, training status, $\dot{V}O_{2\mathrm{peak/max}}$), supplementation protocol (type, dose, frequency, duration, timing of last dose relative to exercise onset, total exposure, placebo, and washout period between trials), exercise protocol (mode, intensity, duration, recovery between bouts, and number of repetitions), and mean ± SD of relevant outcomes, including the mean of all peak power outputs (PP), PP during the first sprint (PP_First_), PP during the last sprint (PP_Last_), time to reach PP (PP_Time_), mean power output from all repetitions (MP), MP during the first sprint (MP_First_), MP during the last sprint (MP_Last_), minimum power (P_Min_), total work performed in repeated cycling efforts (TWD), and total distance covered in the Yo-Yo IR1 running test (TDC). When standard error of the mean (SEM) was reported, SD was calculated as SD = SEM × √*n*, where *n* represents the sample size. Authors of studies included in the meta-analysis were contacted to retrieve individual participants’ data for the calculation of pooled SD and correlation coefficient. For 15 studies, data for individual participants were provided [35,36,37,38,41,51,52,53,54,55,56,57,58,59,60]. The correlation coefficient (Corr) was imputed for the studies with available individual participant data using the following formula:

Corr = SD_E_^2^ + SD_C_^2^ − SD^2^_diff_/2 × SD_E_ × SD_C_,

where: 

Corr = correlation, SD_E_ = standard deviation for the NO_3_^−^ trial, SD_C_ = standard deviation for the placebo trial, SD_diff_ = the difference between the standard deviation for the NO_3_^−^ trial and standard deviation for the placebo trial. 

Subsequently, the standard error of the SMD (SE(SMD)) was calculated using the formula:

SE(SMD) = √1/*n* + SMD^2^/2*n* × √2(1 − Corr),

where:

SE(SMD) = the standard error for the standardised mean difference, *n* = sample size, and Corr = correlation coefficient. 

For the remaining studies (*n* = 10) [34,39,61,62,63,64,65,66,67], Corr was estimated as the average Corr from the studies in which individual data were available.

### 2.5. Quality Assessment 

Risk of bias of included studies was assessed using the Revised Cochrane Collaboration risk of bias tool (ROB2) for crossover trials [68], which assesses studies based on five specific domains: (1) randomisation process; (2) deviations from the intended outcome; (3) missing outcome data; (4) measurement of the outcome; and (5) selection of the reported results. This was performed on the Cochrane excel tool available at https://www.riskofbias.info (accessed on 31 January 2022), which allows an entry for each domain in a risk of bias table rated as “low risk”, “some concerns”, or “high risk”. Two researchers (N.S.A., and A.A.) independently evaluated the risk of bias for each study and any discrepancies were resolved through discussion. As previously recommended [69], funnel plot asymmetry was visually inspected to assess publication bias for meta-analyses that included ≥10 studies.

### 2.6. Statistical Analysis 

Quantitative synthesis was only performed if ≥2 studies measured the same outcome. The meta-analysis was conducted using RevMan 5.4v [70]. A separate meta-analysis was performed for each of the following continuous outcomes: PP, PP_First_, PP_Last_, MP, MP_First_, MP_Last_, PP_Time_, TWD, and TDC. Data are presented as forest plots with 95% confidence intervals. Due to significant between-study heterogeneity, effect sizes were calculated with an inverse variance random-effects model using the DerSimonian–Laird method [71]. Effect sizes were interpreted according to Cohen’s guidelines where an SMD of 0.2, 0.5, and 0.8, respectively, reflect small, medium, and large effects [72]. Heterogeneity was assessed using the Chi^2^ and I^2^ statistics. A value of *p* ≤ 0.10 on the Chi^2^ test was considered significant. The I^2^ was interpreted as follows: <25%, low risk; 25–75%, moderate risk; and >75% high risk [69]. Additionally, forest plots were visually inspected to check for observable differences in study results. A sensitivity analysis was conducted by using a correlation coefficient of 0.5 for all studies [73], removing studies that had a high risk of bias for at least one domain, and those with elite endurance athletes, as previous studies have reported that dietary NO_3_^−^ supplementation is less effective in this population [60,63]. For sub-group analysis, the influence of the NO_3_^−^ supplementation dose (<8 mmol vs. ≥8 mmol) and duration (single day vs. multiple days supplementation), exercise type (single vs. repeated sprints), and exercise duration (≤15 s vs. >15 s–≤30 s) were assessed. Due to the low number of studies that measured plasma [NO_3_^−^] and [NO_2_^−^] and included female participants, a sub-group analysis on the influence of plasma [NO_3_^−^] and [NO_2_^−^] and biological sex could not be performed. Studies recruiting well-trained endurance athletes were omitted from sub-group analyses on the basis that this population group does not exhibit an ergogenic effect after NO_3_^−^ supplementation [60,63]. Statistical significance was accepted at *p* < 0.05. 

## 3. Results

A total of 1538 articles were retrieved from the two databases; after duplicates were removed, 1328 articles remained. No studies were identified through searching the reference lists of included studies. Following initial screening of titles and abstracts, thirty-two full-text articles were retrieved, of which five were excluded for failing to meet the inclusion criteria. Twenty-seven studies were identified as eligible for the systematic review and twenty-five for the meta-analysis. Results of the search strategy are presented in Figure 1.

### 3.1. Study Characteristics 

Table 1 provides a summary of the studies included in the systematic review and meta-analysis. All studies employed a randomised, double (*n* = 23) [34,35,36,37,38,39,40,41,51,53,54,55,56,57,59,60,61,62,64,66,67,74,75] or single (*n* = 4) [52,58,63,65] blind, placebo controlled, crossover design. Studies were published between 2013 and 2022. The sample size varied between studies (range: 7–52 participants). Participants’ ages ranged from 17 to 31 years. Participant training status was described as healthy or recreationally active (*n* = 4) [39,52,57,65], competing at a recreational or amateur standard (*n* = 18) [35,36,37,38,40,41,51,54,55,56,58,59,61,62,66,67,74,75], highly competitive (*n* = 5) [34,36,40,53,64], or elite (*n* = 3) [36,60,63]. Participants were involved in different types of sports, including team sports (*n* = 13) [34,37,38,41,51,52,55,56,62,65,66,74,75], cycling (*n* = 3) [34,36,60], resistance training (*n* = 4) [35,54,57,67], tennis (*n* = 2) [34,40], mixed martial arts (*n* = 1) [64], kayaking (*n* = 1) [53], speed skating (*n* = 1) [36], CrossFit (*n* = 1) [59], and sprinting (*n* = 1) [61]. The dose, duration, and type of NO_3_^−^ supplementation varied between studies. NO_3_^−^ supplementation was administered as beetroot juice (*n* = 24) [34,35,36,37,38,39,40,41,51,53,54,55,56,57,58,60,61,62,63,64,65,66,74,75], potassium NO_3_^−^ (*n* = 1) [59], pomegranate extract (*n* = 1) [67], or as a high NO_3_^−^ diet (*n* = 1) [52]. The dose of NO_3_^−^ supplementation ranged from 4.8 to 16.4 mmol/day (mean; 8.5 mmol/day). Fifteen studies administered NO_3_^−^ supplementation as a single dose 2.5–3 h before exercise [34,35,39,40,53,54,56,57,61,62,64,66,67,74,75] and twelve studies as repeated doses over 2–7 days [36,37,38,41,51,52,55,58,59,60,63,65]. In these latter studies, the last dose was administered 40–180 min before (*n* = 11) [36,37,38,41,51,52,55,58,60,63,65] or ≥24 h before exercise (*n* = 1) [59]. Total NO_3_^−^ exposure in all studies ranged between 4.8 and 77.4 mmol. Most of the included studies recruited exclusively male participants (*n* = 22) [35,37,38,39,40,41,51,52,53,54,55,56,57,58,59,60,61,62,63,64,65,75], four studies recruited male and female participants [34,36,66,67], and one study recruited only female participants [74]. Of the 410 participants included in the review, 354 participants (86%) were reported as male, with 56 participants (14%) reported as female. The most frequent modality of exercise was cycling (*n* = 19) [34,35,36,39,41,51,52,54,56,57,58,59,60,61,63,64,65,66,67], followed by running (*n* = 7) [37,38,40,55,62,74,75] and kayaking (*n* = 1) [53]. Studies used different exercise protocols to assess performance: repeated all-out sprints with a fixed number of repetitions (*n* = 13) [34,36,39,41,51,52,53,56,60,64,67,74,75], high-intensity intervals (*n* = 3) [63,65,66], the 30 s Wingate test (*n* = 7) [34,35,36,54,57,59,61], and the Yo-Yo intermittent recovery level 1 test (Yo-Yo IR1) (*n* = 4) [37,38,55,62]. Different assessment methods were used to evaluate exercise performance, with each study measuring 1–4 performance variables. Performance variables included PP (*n* = 11) [34,35,41,54,56,57,58,59,60,64,66], PP during a single sprint (*n* = 7) [36,39,52,53,60,64,67], time to reach PP (*n* = 4) [35,36,54,57], MP (*n* = 13) [35,41,54,56,57,58,59,60,61,63,64,65,66], MP during a single sprint (*n* = 11) [36,39,41,54,56,57,58,60,61,64,67], TWD (*n* = 6) [34,51,56,61,65,66], minimum power (*n* = 3) [35,54,57], optimal pedalling cadence (*n* = 1) [34], number of completed repetitions (*n* = 3) [63,65,66], TDC (*n* = 4) [37,38,55,62], sprint time (*n* = 3) [40,74,75], best sprint time (*n* = 2) [74,75], slowest sprint time (*n* = 1) [75], and fatigue index (*n* = 6) [34,53,54,57,58,64]. Of the twenty-seven studies included, only eight studies measured plasma [NO_3_^−^] and [NO_2_^−^] [36,37,38,51,52,53,55,65], two studies measured only plasma [NO_3_^−^] [74,75], and one study only measured plasma [NO_2_^−^] [41]. 

### 3.2. Quality Assessment 

**Figure 2:**
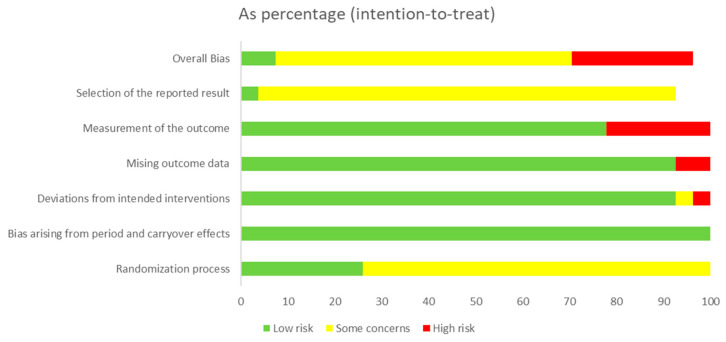
Summary risk of bias graph for crossover trials evaluating the effects of nitrate supplementation on different performance outcomes during single and repeated bouts of short-duration high-intensity exercise.

Five studies had a low risk of bias in the overall bias domain [34,39,54,59,67], fifteen studies had some concerns [35,37,38,40,41,51,53,55,56,61,62,64,66,74,75], and seven studies had a high risk of bias [36,52,57,58,60,63,65]. Seven studies had a low risk of bias in the randomisation process [34,36,39,54,59,63,67] and the remaining twenty studies had some concerns [35,37,38,40,41,51,52,53,55,56,57,58,60,61,62,64,65,66,74,75]. All studies had a low risk of bias for bias arising from period and carryover effects [34,35,36,37,38,39,40,41,51,52,53,54,55,56,57,58,59,60,61,62,63,64,65,66,67,74,75]. Twenty-five studies had low risk of bias [34,35,36,37,38,39,40,41,51,52,53,54,55,56,57,59,60,61,62,63,64,66,67,74,75], one study had a low risk of bias [58], and one study had some concerns [65] in the deviation from the intended intervention domain. For missing outcome data, twenty-five studies had a low risk of bias [34,35,37,38,39,40,41,51,52,53,54,55,56,57,58,59,61,62,63,64,65,66,67,74,75] and two studies had a high risk [36,60]. In the measurement of the outcome domain, six studies had a high risk of bias [52,57,58,60,63,65] and the remaining twenty-one had a low risk of bias [34,35,36,37,38,39,40,41,51,53,54,55,56,59,61,62,64,66,67,74,75]. One study had a low risk of bias [57] and twenty-six studies had some concerns [34,35,36,37,38,39,40,41,51,52,53,54,55,56,58,59,60,61,62,63,64,65,66,67,74,75] in the selection of reported results domain. A summary of risk of bias for crossover trials is presented in Figure 2 and a risk of bias assessment for individual studies is presented in Figure S1 in the online Supplementary Materials. Funnel plots suggest little evidence of publication bias, as presented in the online Supplementary Materials (Figures S2–S5).

### 3.3. Meta-Analysis 

#### 3.3.1. Time to Reach Peak Power 

**Figure 3:**
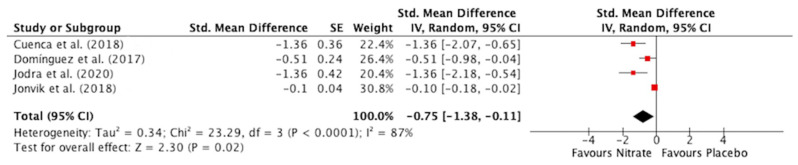
Forest plot for time to reach peak power in the nitrate and placebo trials [35,36,54,57].

NO_3_^−^ supplementation lowered PP_Time_ compared to placebo (SMD: 0.75, 95% CI: −1.38 to 0.11, *p* = 0.02) (Figure 3). There was a high risk of statistical heterogeneity between studies (Chi^2^ = 23.29; I^2^ = 87%, *p* < 0.0001). Removing a study with a high risk of bias [57] did not remove statistical heterogeneity but slightly changed the pooled SMD (SMD: 0.88, 95% CI: −1.90 to 0.13, *p* = 0.09). 

#### 3.3.2. Peak Power 

There was no difference between dietary NO_3_^−^ and placebo supplementation in PP (SMD: 0.01, 95% CI: −0.06 to 0.08, *p* = 0.75) (Figure S6a), PP_First_ (SMD: 0.05, 95% CI: −0.05 to 0.15, *p* = 0.36) (Figure S6b), and PP_Last_ (SMD: 0.10, 95% CI: −0.06 to 0.27, *p* = 0.23) (Figure S6c). 

#### 3.3.3. Mean Power 

**Figure 4:**
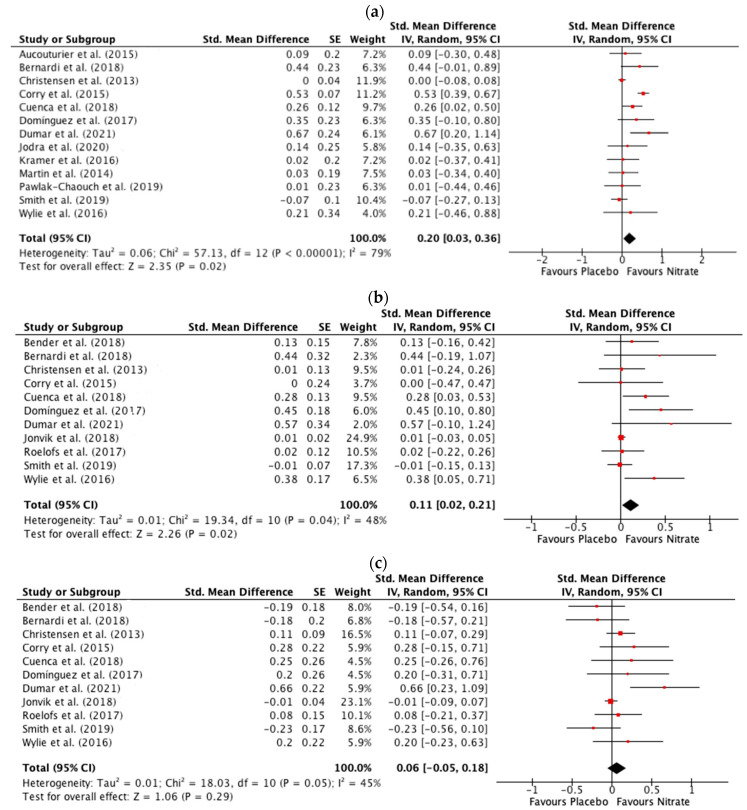
Forest plot for mean power from all sprints (**a**), mean power during the first sprint (**b**), and mean power during the last sprint (**c**) in the nitrate and placebo trials [35,36,39,41,54,56,57,58,59,60,61,63,64,65,66,67].

Both MP (SMD: 0.20, 95% CI: 0.03 to 0.36, *p* = 0.02) (Figure 4a) and MP_First_ (SMD: 0.11, 95% CI: 0.02 to 0.21, *p* = 0.02) (Figure 4b) were greater after dietary NO_3_^−^ compared to placebo supplementation, with no significant difference between dietary NO_3_^−^ and placebo supplementation in MP_Last_ (SMD: 0.06, 95% CI: −0.05 to 0.18, *p* = 0.29) (Figure 4c). There was a high risk of statistical heterogeneity between studies (Chi^2^ = 57.13; I^2^ = 79%, *p* < 0.00001) measuring MP. Sensitivity analyses revealed that excluding studies in elite athletes [60,63] slightly increased the pooled SMD (SMD: 0.24, 95% CI: 0.06 to 0.42, *p* = 0.009) and reduced the statistical heterogeneity (Chi^2^ = 32.89; I^2^ = 70%, *p* < 0.0003), while excluding studies with a high risk of bias [57,58,60] slightly reduced statistical heterogeneity (Chi^2^ ≤ 31.44; I^2^ = 71%, *p* < 0.0002) and the pooled SMD (SMD: 0.18, 95% CI: −0.01 to 0.36, *p* = 0.07). When the influence of NO_3_^−^ dose was isolated, MP was greater after NO_3_^−^ compared to placebo supplementation with high NO_3_^−^ doses ≥ 8 mmol (SMD: 0.27, 95% CI: 0.01 to 0.54, *p* = 0.04), but there were no differences between NO_3_^−^ and placebo supplementation when a NO_3_^−^ dose < 8 mmol was administered (SMD: 0.19, 95% CI: −0.02 to 0.40, *p* = 0.08) (Figure S7a). There was no difference in MP between NO_3_^−^ and placebo supplementation when a single-day supplementation protocol was adopted (SMD: 0.12, 95% CI: −0.03 to 0.26, *p* = 0.11), but the increase in MP after NO_3_^−^ compared to placebo supplementation approached statistical significance when multiple-day supplementation was adopted (SMD: 0.27, 95% CI: 0.01 to 0.54, *p* = 0.05) (Figure S7b). When the influence of exercise type and duration was evaluated, MP was improved after NO_3_^−^ compared to placebo supplementation during a single sprint (SMD: 0.31, 95% CI: 0.10 to 0.51, *p* = 0.004), but not during repeated sprints (SMD: 0.14, 95% CI: −0.04 to 0.32, *p* = 0.13) (Figure S7c) and when sprint time was >15 s–≤30 (SMD: 0.31, 95% CI: 0.12 to 0.50, *p* = 0.001), but not when sprint time ≤ 15 s (SMD: 0.14, 95%, CI: −0.05 to 0.34, *p* = 0.15) (Figure S7d). There were no differences in any of these comparisons for MP_First._

#### 3.3.4. Total Work Done 

**Figure 5:**
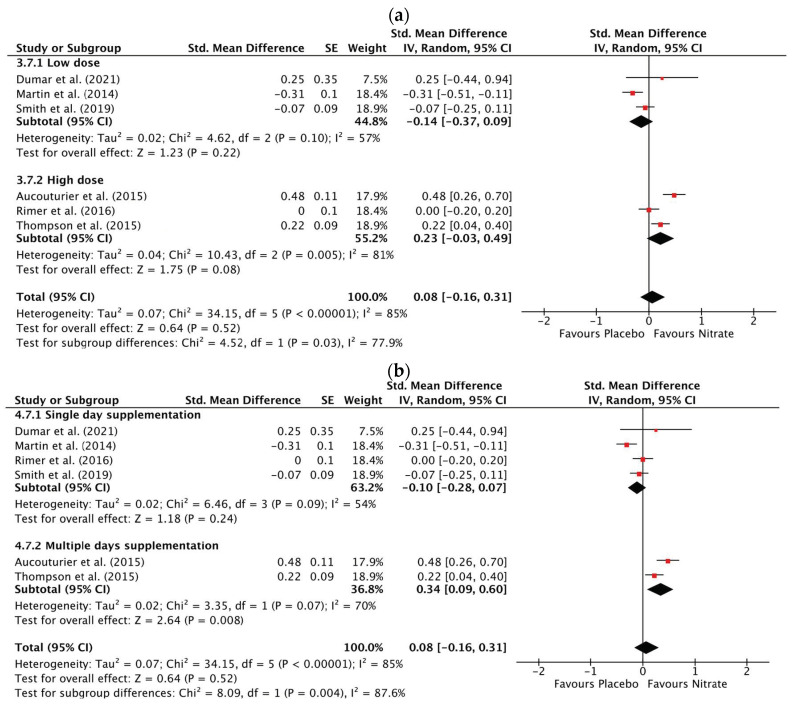
Forest plot of total work performed in the nitrate and placebo trials (**a**), low nitrate dose (<8 mmol/day) compared to high nitrate dose (≥8 mmol/day) (**b**), multiday nitrate supplementation compared to single day nitrate supplementation [34,51,56,61,65,66].

NO_3_^−^ supplementation did not alter TWD compared to placebo (SMD: 0.06, 95% CI: −0.13 to 0.26, *p* = 0.52) (Figure S8). There was a high risk of statistical heterogeneity between studies (Chi^2^ = 34.40; I^2^ = 85%, *p* < 0.00001). Sensitivity analyses did not remove statistical heterogeneity or change the pooled SMD. Sub-group analysis on supplementation dose revealed a significant sub-group difference (*p* = 0.03) between high NO_3_^−^ doses ≥ 8 mmol (SMD: 0.23, 95% CI: −0.03 to 0.49, *p* = 0.08) and low NO_3_^−^ doses < 8 mmol (SMD: −0.14, 95% CI: −0.37 to 0.09, *p* = 0.22) (Figure 5a). The sub-group analysis on supplementation duration revealed a significant difference (*p* = 0.004) between multiple-day supplementation (SMD: 0.34, 95% CI: 0.09 to 0.60, *p* = 0.008) and single-day supplementation (SMD: −0.10, 95% CI: −0.28 to 0.07, *p* = 0.24) (Figure 5b). 

#### 3.3.5. Total Distance Covered 

**Figure 6:**
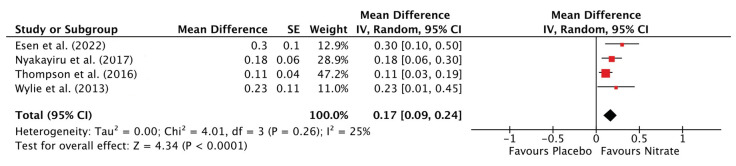
Forest plot for total distance covered in the nitrate and placebo trials [37,38,55,62].

NO_3_^−^ supplementation increased TDC compared to placebo (SMD: 0.17, 95% CI: 0.09 to 0.24, *p* < 0.0001) (Figure 6). There was a low risk of statistical heterogeneity between studies (Chi^2^ = 4.01; I^2^ = 25%, *p* = 0.26). Sub-group and sensitivity analyses could not be performed due to an insufficient number of studies measuring TDC (*n* = 4). 

## 4. Discussion

The principal observations of this systematic review and meta-analyses are that, compared to a placebo condition, NO_3_^−^ supplementation lowered PP_Time_ without impacting PP, increased MP and MP_First_, and increased TDC in the Yo-Yo IR1 test. The improvement in MP after NO_3_^−^ supplementation was more likely to occur when NO_3_^−^ was administered for multiple days at a dose ≥ 8 mmol as opposed to an acute serving of <8 mmol during a single bout rather than repeated bouts of high-intensity exercise, and when the high-intensity exercise duration was >15 s–≤30 s versus ≤15 s. The sub-group analysis also revealed that NO_3_^−^ supplementation was more likely to improve TWD in a high-intensity repeated bout protocol when NO_3_^−^ was administered at a dose ≥ 8 mmol and was supplemented for multiple days as opposed to an acute serving or a dose < 8 mmol. These observations improve our understanding of the effects of NO_3_^−^ supplementation on single and repeated bouts of short-duration, high-intensity, large muscle mass exercise, and reveal two apparently distinct and supplementation-strategy-dependent effects of dietary NO_3_^−^ on high-intensity exercise performance. Firstly, NO_3_^−^ supplementation appears to improve PP_Time_ and MP_First_, with the improvements in these variables not necessarily requiring multiple-day supplementation with ≥8 mmol NO_3_^−^, as such effects appear to be achievable after acute supplementation with ~6 mmol NO_3_^−^. Secondly, TWD in a repeated sprint protocol was more likely to be improved when NO_3_^−^ was administered at a dose ≥ 8 mmol, and was supplemented for multiple days, consistent with the NO_3_^−^ supplementation regime administered in the studies assessing TDC in the Yo-Yo IR1 test, all of which reported improved performance. Therefore, it appears that a single bout of high-intensity exercise can be enhanced by acute NO_3_^−^ supplementation, with high-intensity intermittent exercise performance more likely to improve after multiple day supplementation, with a NO_3_^−^ dose ≥8 mmol. These findings may have implications for future study design and for improving performance in athletes participating in sports that require high-intensity bouts of exercise. 

Although there are some examples of enhanced PP after NO_3_^−^ supplementation [35,52,54,57,58,59,67], the current meta-analysis indicates that most previous studies did not report improved PP, PP_First_, or PP_Last_ after NO_3_^−^ supplementation [36,39,41,53,56,60,64,66]. However, whilst PP was not altered, PP_Time_ was lowered after NO_3_^−^ supplementation with all four studies assessing this variable observing a lower PP_Time_ after NO_3_^−^ supplementation [35,36,54,57], with three of these studies administering an acute NO_3_^−^ dose of ~6 mmol [35,54,57]. This observation is compatible with an increase in muscle contractile velocity, which would be expected to contribute to lower PP_Time_ after acute NO_3_^−^ supplementation [33,34]. With regard to MP variables, MP and MP_First_, but not MP_Last_, were improved after NO_3_^−^ supplementation. When the improvement in MP after NO_3_^−^ supplementation was explored further, MP was improved after NO_3_^−^ supplementation when doses ≥ 8 mmol were administered [41,64,65], when multiple day supplementation protocols were adopted [41,64,65], and when a single sprint >15 s–≤30 s was performed [35,54,58,59]. The improvements in PP_Time_ and MP_First_ were exhibited after acute supplementation with ~6 mmol NO_3_^−^ [35,39,54,57]. All four studies assessing the effect of NO_3_^−^ supplementation on TDC in the Yo-Yo IR1 test revealed a greater TDC after NO_3_^−^ supplementation [37,38,55,62]. While TWD during high-intensity intermittent exercise was not improved after NO_3_^−^ supplementation, the sub-group analysis revealed that TWD was increased when the NO_3_^−^ dose was ≥8 mmol compared to <8 mmol [51,65], and with multiple-day supplementation compared to acute supplementation [51,65]. Importantly, the four studies reporting improved TDC in the Yo-Yo IR1 test all adopted a multiple-day supplementation protocol with a NO_3_^−^ dose of >8 mmol [37,38,55,62]. Therefore, it appears that a multiple-day supplemental protocol with a NO_3_^−^ dose of >8 mmol is important to elicit an ergogenic effect on repeated bouts of high-intensity exercise after NO_3_^−^ supplementation but that performance in single sprints (lower PP_Time_ and higher MP) can be enhanced after acute ingestion of ~6 mmol NO_3_^−^.

The ergogenic effect of NO_3_^−^ supplementation has been attributed to its stepwise reduction to NO_2_^−^ and the subsequent reduction of NO_2_^−^ to NO [2,5]. It is now recognised that ~25% of ingested NO_3_^−^ is extracted from the circulation by the salivary glands [76] via the NO_3_^−^/H^+^ cotransporter, sialin [77]. NO_3_^−^ is subsequently concentrated within salivary glands [78] with excreted salivary NO_3_^−^ undergoing reduction to NO_2_^−^ by certain species of the oral micobiome [79,80,81]. NO_2_^−^-rich saliva is then swallowed and subsequently reduced to NO and various reactive nitrogen intermediates, including S-nitrosothiols (RSNO) within the stomach [2,78], but it is also clear that circulating plasma [NO_2_^−^] and [RSNO] are increased post NO_3_^−^ supplementation [78,82,83]. Circulating plasma NO_2_^−^ can undergo a one-electron reduction to NO in a reaction catalysed by numerous NO_2_^−^ reductases [84,85]. Although the relationship between exercise performance and plasma [NO_3_^−^] is unclear, exercise responses are positively associated with the increases in plasma [NO_2_^−^] [82,86], muscle [NO_3_^−^], and muscle NO_3_^−^ utilisation [87] after NO_3_^−^ supplementation. 

It is increasingly appreciated that skeletal muscle can serve as an important store of NO_3_^−^ and NO_2_^−^ for subsequent NO synthesis, as evidenced by higher [NO_3_^−^] and [NO_2_^−^] in skeletal muscle than blood [88,89]. The NO_3_^−^ transporter, sialin, has been identified in skeletal muscle [89,90] which, together with chloride channel 1 [90], facilitate the concentration of NO_3_^−^ within skeletal muscle. Therefore, a portion of the increased circulating blood NO_3_^−^ after NO_3_^−^ supplementation, which is not extracted by the kidney for clearance in the urine or absorbed by the salivary glands for subsequent oral reduction to NO_2_^−^, can be accrued in skeletal muscle. Indeed, skeletal muscle [NO_3_^−^] and [NO_2_^−^] are increased following NO_3_^−^ supplementation with duration-dependent increases at least up to 7 days of supplementation [88]. In addition to its role as a NO_2_^−^ reductase [91], xanthine oxidoreductase (XOR) can function as a NO_3_^−^ reductase to increase NO_2_^−^ synthesis [92] and is present in skeletal muscle [89,90]. It has been reported that the increase in skeletal muscle [NO_2_^−^] after NO_3_^−^ administration is enhanced by exercise and, as muscle pH is lowered, with both NO_3_^−^ reduction to NO_2_^−^ and NO_2_^−^ reduction to NO abolished after XOR inhibition [93]. It is, therefore, possible that increased XOR activity during exercise, particularly high-intensity exercise [94], could contribute to enhanced muscle NO_3_^−^ and NO_2_^−^ reduction in such settings. Indeed, the increase in skeletal muscle [NO_3_^−^] after NO_3_^−^ supplementation is lowered following the completion of exhaustive cycling exercise [89] and maximal knee extensor contractions [87], suggesting that this elevated muscle NO_3_^−^ pool is utilised as a substrate for sequential reduction to NO_2_^−^ and then NO. There is also a positive arterial-venous difference in plasma [NO_3_^−^] and [NO_2_^−^] across contracting skeletal muscles after NO_3_^−^ supplementation [95]. Since NO_2_^−^ reduction to NO is augmented in hypoxia and acidosis [26,27,28], and given that such conditions develop within the muscle microvasculature during exercise in an intensity-dependent manner [31], elevating circulating plasma [NO_2_^−^] is likely to increase NO synthesis in the muscle microvasculature during high-intensity exercise. Based on the existing evidence, NO_3_^−^ and NO_2_^−^ can be increased systemically and within skeletal muscle following dietary NO_3_^−^ supplementation with the potential to enhance NO synthesis, particularly during the hypoxic and acidic conditions that develop during high-intensity exercise, which might underpin the improvements in high-intensity exercise performance variables reported in this manuscript.

The improvements in PP_Time_ and MP_First_ during an all-out sprint after NO_3_^−^ supplementation are likely mediated by mechanisms intrinsic to the myocytes. The initial stages of a short-duration all-out sprint, during which PP_Time_ will be determined, will involve maximal recruitment of, and proportion contribution to force production from, type II skeletal muscle fibres [96,97]. Previous research has indicated that 7 days NO_3_^−^ supplementation can increase calcium (Ca^2+^) handling proteins and evoke force production in type II skeletal muscle, but not slow-twitch (type I) skeletal muscle, in mice [18]. However, three [35,54,57] of the four [35,36,54,57] studies reporting improved PP_Time_, and six [39,54,56,57,64,67] of the eleven [36,39,41,54,56,57,58,60,61,64,67] studies reporting improved MP_First_ after NO_3_^−^ supplementation administered NO_3_^−^ acutely, and it has been reported that increased evoked muscle force production can occur independently of changes in Ca^2+^ handling proteins in human skeletal muscle [98]. Therefore, the improvements in PP_Time_ and MP_First_ after NO_3_^−^ supplementation are likely to be underpinned by NO-cyclic guanosine monophosphate (cGMP)-mediated signalling and/or post-translational modification of protein thiols [99]. 

In contrast to the NO_3_^−^ supplementation regime required to improve PP_Time_ and MP_First_, TWD during high-intensity intermittent exercise was improved after NO_3_^−^ supplementation when the NO_3_^−^ dose was ≥8 mmol, but not <8 mmol, and only with multiple-day supplementation. There was also a greater TDC in the Yo-Yo IR1 after NO_3_^−^ supplementation with all studies reporting this ergogenic effect employing multiple-day NO_3_^−^ supplementation at a daily dose ≥8 mmol. Greater ergogenic effects during high-intensity intermittent exercise after multiple-day, higher dose NO_3_^−^ supplementation might be linked to the greater time course to increase muscle [NO_2_^−^] after NO_3_^−^ supplementation as, unlike muscle [NO_3_^−^], muscle [NO_2_^−^] is not increased after acute NO_3_^−^ ingestion [87,89] but can be increased after 7 days of NO_3_^−^ ingestion [88]. Indeed, when mouse single myocytes were acutely exposed to increased NO_2_^−^, contractile function and Ca^2+^ handling were not altered in the earlier stages of a fatigue-inducing contraction protocol, whereas time to task failure was extended as a result of better maintenance of myocyte contractility, Ca^2+^ sensitivity, and Ca^2+^ pumping towards the latter stages of the protocol [100]. In human skeletal muscle, greater potential for improved muscle contractile responses during a fatigue-inducing 60 maximum voluntary contraction protocol has been reported during the initial contractions after acute NO_3_^−^ ingestion [87] and following completion of the fatiguing protocol after multiple-day NO_3_^−^ supplementation [101]. Skeletal muscle [NO_3_^−^] and [NO_2_^−^] increase in a duration-dependent manner following NO_3_^−^ supplementation [88], and muscle [NO_3_^−^] declines during sustained high-intensity exercise [87,89] and is correlated with improved muscle force production [87]. Therefore, multiple-day NO_3_^−^ supplementation with a NO_3_^−^ dose exceeding 8 mmol may be more effective at improving MP during a single 15–30 s bout of high-intensity exercise or at improving TWD or TDC during high-intensity intermittent exercise by eliciting greater increases in muscle [NO_3_^−^] and [NO_2_^−^] to support greater NO_3_^−^ reduction and NO generating potential during these high-intensity exercise settings. As such, NO_3_^−^ may impact skeletal muscle contractile function in a supplementation-strategy-dependent manner that may be mediated by different muscle exposures to NO_3_^−^ and NO_2_^−^. 

Although the findings of the current study may have implications for improving NO_3_^−^ supplementation strategies to bolster performance in different types of high-intensity exercise, there are several limitations of, and experimental considerations from, the studies included in this systematic review and meta-analysis. Firstly, the SMD was typically small across all variables that did exhibit an ergogenic effect after NO_3_^−^ supplementation, which underscores the importance of assessing the translational potential of these findings to improve in-competition performance in sports where performance outcomes are dictated by the capability to perform high-intensity exercise. Moreover, the meta-analysis conducted on PP_Time_ and MP exhibited high heterogeneity, indicating a substantial variation in the results of the included studies. Since a limited number of studies assessed plasma [NO_3_^−^] and [NO_2_^−^] and included female participants, not all planned sub-analyses could be completed. There was also limited assessment of the physiological mechanisms for any improvement in high-intensity exercise performance in the studies included in the current systematic review and meta-analyses. Therefore, further research is required to resolve the putative mechanisms for improved performance during single and repeated bouts of short duration high-intensity exercise and the extent to which such mechanisms are influenced by acute and multiple-day NO_3_^−^ ingestion and mediated by plasma and muscle [NO_3_^−^] and [NO_2_^−^] and different population groups

## 5. Conclusions

The current study conducted a systematic review and completed several meta analyses to evaluate the effect of dietary NO_3_^−^ supplementation of different aspects of high-intensity exercise performance, with sub-analyses conducted to provide wider contextual insight. It was observed that NO_3_^−^ supplementation lowered PP_Time_, increased MP and MP_First_, and increased TDC in the Yo-Yo IR1 test, supporting the ergogenic potential of dietary NO_3_^−^ supplementation for some aspects of high-intensity exercise performance. Sub-group analyses revealed that MP was more likely to be improved during a single >15 s–≤30 s versus ≤15 s bout rather than repeated bouts of high-intensity exercise, and that MP, TWD, and TDC were more likely to be improved after multiple-day supplementation with a daily NO_3_^−^ dose ≥8 mmol compared to acute ingestion of <8 mmol NO_3_^−^. These findings improve our understanding of the ergogenic potential of dietary NO_3_^−^ supplementation for high-intensity exercise and can help inform NO_3_^−^ supplementation strategies to improve high-intensity exercise performance. 

## Figures and Tables

**Table 1:** Summary of studies included in the systematic review and meta-analysis that examined the effects of nitrate supplementation on exercise performance during single and repeated bouts of short duration high-intensity exercise.

Study	Participants			Supplementation Protocol							
	No. (♂, ♀)	Health/Training Status	Age (Years)	Type/Volume	NO_3_^−^ Dose (mmol)	Duration	Time before Trial	Placebo	Exercise Protocol	Performance Variables	Results
Aucouturier et al. (2015) [65]	17 ♂	Healthy, active in team sports	23 ± 3	BR juice/500 mL	10.9	3 D	3 h	Apple-black currant juice	15 s cycling at 170% of MAP to exhaustion, interspersed with 30 s	MP, TWD, reps, exercise duration	ND in MP, improved TWD, reps and exercise duration
Bender et al. (2018) [39]	16 ♂	Healthy, recreationally active	17 ± 1	BR shot/2 × 70 mL	12.9	Single D	3 h	NR-depleted BR shot	4 × 20 s all-out WAnT, interspersed with 240 s	PP, MP	ND in PP and MP
Bernardi et al. (2018) [64]	10 ♂	Well-trained mixed martial arts athletes	25 ± 5	BR juice/400 mL	9.3	Single D	2 h	Black current juice	20 × 6 s all-out cycling interspersed with 24 s	PP, MP, FI	ND in PP, MP, and FI
Buck et al. (2015) [74]	13 ♀	Team sport players	26 ± 2	BR shot/1 × 70 mL	6	Single D	3 h	NR-depleted BR shot	6 × 20 m all-out effort running, interspersed with 25 s recovery	ST, best ST	ND in ST and best ST
Christensen et al. (2013) [60]	10 ♂	Elite cyclists	29 ± 4	BR juice/500 mL	8	4 D	3 h	Apple-black currant juice	6 × 20 s cycling at 0.75 N/kg, interspersed with 100 s	PP, MP	ND in PP and MP
Corry et al. (2015) [58]	10 ♂	Recreationally active	20 ± 1	BR shot/2 × 70 mL	8	2 D	40 min	Black current juice	30 s all-out WanT	PP, MP, FI	Improved MP, ND in PP and FI
Cuenca et al. (2018) [54]	15 ♂	Resistance trained	22 ± 2	BR shot/1 × 70 mL	6	Single D	3 h	NR-depleted BR juice	30 s all-out WAnT	PP, MP, PP_Time_, P_Min_, FI	Improved PP, MP and PP_Time_, ND in FI
Domínguez et al. (2017) [57]	15 ♂	Healthy trained	22 ± 2	BR shot/1 × 70 mL	5.6	Single D	3 h	NR-depleted BR juice	30 s all-out WAnT	PP, MP, PP_Time_, P_Min_, FI	Improved PP and MP, ND in PP_Time_, P_Min_, FI
Dumar et al. (2021) [61]	10 ♂	National level sprinters	20.3 ± 2	BR shot/1 × 70 mL	6.4	Single D	2 h	Black current juice	3 × 15 s all-out WAnT	MP and TWD	Improved MP and TWD
Esen et al. (2022) [62]	12 ♂	Recreational active	27 ± 10	BR shot/1 × 140 mL	12.8	Single D	3 h	BR shot/1 × 70 mL	Yo-Yo IR1 test	TDC	Longer TDC
Jodra et al. (2020) [35]	15 ♂	Resistance trained	23 ± 2	BR shot/1 × 70 mL	6.4	Single D	2.5-3 h	NR-depleted BR juice	30 s all-out WAnT	PP, MP, PP_Time_,P_Min_	Improved PP and PP_Time_, ND in MP and P_Min_
Jonvik et al. (2018) [36]	29 ♂ 23 ♀	Recreational cyclists (*n* = 20), national talent speed skaters (*n* = 23), Olympic- level track cyclists (*n* = 10)	♂ = 22 ± 5 ♀ = 26 ± 8	BR shot/2 × 70 mL	12.9	6 D	3 h	NR-depleted BR juice	3 × 30 s all-out WAnT interspersed with 240 s recovery	PP, MP, PPTime	ND in PP and MP, improved PPTime
Kramer et al. (2016) [59]	12 ♂	CrossFit athletes	23 ± 5	KNR/2 capsules	8	6 D	≥24 h	KCL capsules	30 s all-out WAnT	PP, MP	Improved PP, ND in MP
López-Samanes et al. (2020) [40]	13 ♂	Highly competitive tennis players	25 ± 5	BR shot/1 × 70 mL	6.4	Single D	3 h	NR-depleted BR juice	10 m Sprint	ST	ND in ST
Martin et al. (2014) [66]	9 ♂ 7 ♀	Moderately trained team sport athletes	♂ = 22 ± 2 ♀ = 21 ± 1	BR shot/1 × 70 mL	4.8	Single D	2 h	NR-depleted BR shot	8 s high intensity cycling to exhaustion interspersed with 30 s	PP, MP, TWD, no of reps	ND in PP, MP, TWD, no of reps
Muggeridge et al. (2013) [53]	8 ♂	Trained kayakers	31 ± 15	BR shot/1 × 70 mL	5	Single D	3 h	Tomato juice	5 × 10 s maximum effort kayaking, interspersed with 50 s recovery	PP, FI	ND in PP and FI
Nyakayiru et al. (2017) [55]	32 ♂	Soccer players	23 ± 1	BR shot/2 × 70 mL	12.9	6 D	3 h	NR-depleted BR shot	Yo-Yo IR1 test	TDC	Longer TDC
Pawlak-Chaouch et al. (2019) [63]	11 ♂	Elite endurance athletes	22 ± 4	BR juice/500 mL	5.5	3 D	3 h	Apple-black currant juice	15 s cycling at 170% of MAP to exhaustion interspersed with 30 s	MP, TWD and no of reps	ND in MP, TWD and no of reps
Porcelli et al. (2016) [52]	7 ♂	Healthy recreationally active	25 ± 2	High NR diet	8.2	6 D	3 h	Control diet ~2.9 mmol NR/day	5 × 6 s all-out cycling, interspersed with 24 s recovery	PP	Improved PP
Reynolds et al. (2020) [75]	16 ♂	Team sport athletes	21 ± 2	BR shot/1 × 70 mL	6	Single D	3 h	NR-depleted BR shot	10 × 40 m all-out running interspersed with 30 s recovery	ST, fastest ST, slowest ST	ND in ST, fastest ST and slowest ST
Rimer et al. (2017) [34]	11 ♂ 2 ♀	Competitively trained athletes	26 ± 8	BR shot/2 × 70 mL	11.2	Single D	2.5 h	NR-depleted BR shot	4 × 3–4 s all-out cycling interspersed with 120 s. Followed by 30 s WAnT after 300 s rest.	PP, TW, optimal pedalling rate, FI	Improved PP and optimal pedalling rate during 4 × 3–4 s test. ND in PP, TW, and FI during 30 s Wingate test
Roelofs et al. (2017) [67]	10 ♂ 11 ♀	Recreationally resistance-trained	22 ± 2	Pomegranate extract/capsule	6.8	Single D	-	Maltodextrin capsule	10 × 6 s all-out, interspersed with 30 s	PP, MP	Improved PP and MP
Smith et al. (2019) [56]	12 ♂	Recreationally trained, team sport athletes	22 ± 4	BR shot/1 × 70 mL	6.2	Single D	2.5 h	NR-depleted BR shot	2 halves of 20 × 6 s all out cycling interspersed with 114 s recovery	PP, MP, TWD	ND in PP, MP, TW
Thompson et al. (2016) [37]	32 ♂	Team-sport players	24 ± 4	BR shot/1 × 70 mL	6.4	5 D	2.5 h	NR-depleted BR shot	Yo-Yo IR1	TDC, 20 m sprint time, 5, 10, 5–10, 10–20 m split time	Longer TDC, improved 5, 10, 5–10 m split time, ND in 10-20 m split time
Thompson et al. (2015) [51]	16 ♂	Recreational team-sport players	24 ± 5	BR shot/2 × 70 mL	12.8	7 D	2.5 h	NR-depleted BR shot	2 halves of 20 × 6 s all out cycling interspersed with 114 s recovery	TWD	Improved TWD
Wylie et al. (2016) [41]	10 ♂	Recreational team-sport players	21 ± 1	BR shot/2 × 70 mL	8.2	3 D	2.5 h	NR-depleted BR shot	D3: 24 × 6 s all out cycling interspersed with 24 s D4: 7 × 30 s all-out cycling interspersed with 240 s D5: 6 × 60 s interspersed with 60 s	PP, MP	Improved PP and MP during 24 × 6 s. ND in PP and MP during 7 × 30 s and 6 × 60 s
Wylie et al. (2013) [38]	14 ♂	Recreational team-sport players	22 ± 2	Day 1, BR shot/4 × 70 mL Day 2, BR shot/3 × 70 mL	D1:16.4 D2:12. 3	2 D	1.5 h	NR-depleted BR shot	Yo-Yo IR1	TDC	Longer TDC

NR, nitrate; BR, beetroot; PL, placebo; PP, peak power; PP_Time_, time to peak power; MP, mean power output; P_Min_, minimum power; TWD, total work done; TDC, total distance covered; ST; sprint time; TT; time trial; reps, number of repetitions; FI, fatigue index; MAP, maximal aerobic power, HI, high intensity; Yo-Yo IR1; Yo-Yo intermittent recovery level 1 test; WAnT. Wingate anaerobic test; resistance, kg; kilograms; D, day; h, hour; s, second; min, minutes; ND, no difference; KNR, potassium nitrate; KCL, potassium chloride; -, no information provided; no., number of participants; ♂, male biological sex; ♀, female biological sex.

## Data Availability

Data can be provided at reasonable request from corresponding authors.

## Supplementary Materials

The following supporting information can be downloaded at: https://www.mdpi.com/article/10.3390/antiox12061194/s1: Search strategy; Table S1: Population, Intervention, Comparator, Outcome, Study design (PICOS) framework for study eligibility; Figure S1: Risk of bias summary for individual for crossover trials; Figure S2: Funnel plot evaluating publication bias of trials assessing mean peak power output (*n* = 12); Figure S3: Funnel plot evaluating publication bias of trials assessing mean of the mean power output (*n* = 12); Figure S4: Funnel plot evaluating publication bias of trials assessing mean power output during the first sprint (*n* = 10); Figure S5: Funnel plot evaluating publication bias of trials assessing mean power output during the last sprint (*n* = 10); Figure S6: Forrest plot for mean peak power output (a), peak power during the first sprint (b), and peak power during the last sprint (c) in the nitrate and placebo trials; Figure S7: Forrest plot for mean power output sub-group analyse; low nitrate dose <8 mmol compared to high nitrate dose ≥8 mmol (a), single day nitrate supplementation compared multiple days nitrate supplementation (b), single sprint compared to repeated sprints (c), exercise duration ≤ 15 s compared to exercise duration >15 s–≤30 s (d); Figure S8: Forrest plot for total work done in the nitrate and placebo trials.
