# Supplementary material for: Effects of Dietary Nitrate Supplementation on Performance during Single and Repeated Bouts of Short-Duration High-Intensity Exercise: A Systematic Review and Meta-Analysis of Randomised Controlled Trials
Source: Antioxidants (Basel). 2023 May 31;12(6):1194. doi: 10.3390/antiox12061194 (PMC10294948; doi:10.3390/antiox12061194)
Supplement: Supplementary file 1 [file antioxidants-12-01194-s001.zip › antioxidants-2422204-Suppllemntary Materials..pdf]

Online supplementary materials

Search Strategy (Medline)

S1. nitrate* (76,814)

S2. nitrite* (40,613)

S3. beetroot (705)

S4. (MH "Beta Vulgaris") (2,035)

S5. spinach (8,470)

S6. high nitrate diet (40)

S6. S1 OR S2 OR S3 OR S4 OR S5 (110,132)

S7. TX exercise performance (12,999)

S8. high-intensity intermittent exercise (1,231)

S9. high intensity exercise (5,430)

S10. supramaximal exercise (317)

S11. intermittent exercise (2,356)

S12. sprint* (8,733)

S13. all out (28,433)

S14. repeated sprint* (1,155)

S15. run* (220,658)

S16. cycl* (1,387,868)

S17. row* (75,488)

S18. TX "power output" (8,003)

S19. TX "distance covered" (1,374)

S20. TX "completion time" (1,520)

S21. TX "work rate" (2,668)

S22. fatigue* (108,075)

S23. wingate test (1,026)

S24. yo-yo test (448)

S25. S7 OR S8 OR S9 OR S10 OR S11 OR S12 OR S13 OR S14 OR S15 OR S16 OR S17 OR S18 OR S19 OR S20 OR S21 OR S22 OR S23 OR S24 (1,804,060)

S26. S6 AND S25 (12,932)

S27. (MH "Randomized Controlled Trial") OR (MH "Controlled Clinical Trial") OR (MH "Controlled Clinical Trials as Topic") OR (MH "Randomized Controlled Trials as Topic") OR (MH "Random Allocation") (245,099)

S28. TX randomly (335,141)

S29. TX randomi?ed (897,912)

S30. AB placebo (208,574)

S31. AB trial (894,619)

S32. S27 OR S28 OR S29 OR S30 OR S31 (1,656,249)

S33. S32 AND S26 **(823)**

Search Strategy (Sportdiscus)

S1. TX nitrate* (3,234)

S2. TX nitrite* (1,787)

S3. TX beetroot (598)

S4. TX beet root (105)

S5. TX beet (2,677)

S6. TX beta Vulgaris (53)

S7. TX spinach (3,631)

S8. TX performance enhancer (648)

S9. high nitrate diet (21)

S10. S1 OR S2 OR S3 OR S4 OR S5 OR S6 OR S8 OR S9 (10,293)

S11. exercise performance (8,700)

S12. high intensity intermittent exercise (421)

S13. high intensity exercise (3,970)

S14. supramaximal exercise (357)

S15. intermittent exercise (1,790)

S16. repeated sprint* (1,445)

S17. run* (140,325)

S18. cycl* (106,843)

S19. row* (20,564)

S20. all out (2,670)

S21. wingate test (1,604)

S22. sprint* (21,758)

S23. fatigue* (23,558)

S24. TX "power output" (11,976)

S25. TX "distance covered" (3,550)

S26. TX "completion time" (1,028)

S27. TX "work rate" (4,033)

S28. yo-yo test (577)

S29. S11 OR S12 OR S13 OR S14 OR S15 OR S16 OR S17 OR S18 OR S19 OR S20 OR S21 OR S22 OR S23 OR S24 OR S25 OR S26 OR S27 OR S28 (306,659)

S30. S29 AND S10 (1,629)

S31. TX randomi?ed control* trial* (52,723)

S32. randomi?ed N3 study (8,440)

S33. placebo N3 study (2,238)

S34. random* N7 order* (3,776)

S35. cross#over* OR cross N1 over* (8,443)

S36. TX randomly (65,616)

S37. AB placebo (13,433)

S38. AB trial (56,625)

S39. S30 OR S31 OR S32 OR S33 OR S34 OR S35 OR S36 OR S37 (150,141)

S40. S30 AND S39 **(510)**

Table S1: Population, Intervention, Comparator, Outcome, Study design (PICOS) framework for study eligibility.

**PICOS methodology**		**Corresponding search terms**
**Population**	Healthy young adult participants ≥16 years old	Humans
**Intervention**	Inorganic nitrate or nitrite supplementation as beetroot juice, sodium/potassium nitrate, or nitrate rich diet.	Nitrate-rich beetroot juice, nitrate-rich diet, sodium nitrate, potassium nitrate
**Comparator**	Placebo or control group receiving nitrate-depleted compounds or foods.	Placebo, control, nitrate-depleted beetroot juice, low nitrate diet, sodium chloride, potassium chloride
**Outcome**	Trials reporting effects of nitrate supplementation on exercise performance	Total distance covered, maximal/mean power output, total/maximal work rate, completion time
**Study design**	Randomised controlled trials	Placebo-controlled trial<br>randomized controlled trial<br>Randomised/randomized<br>randomly<br>trials

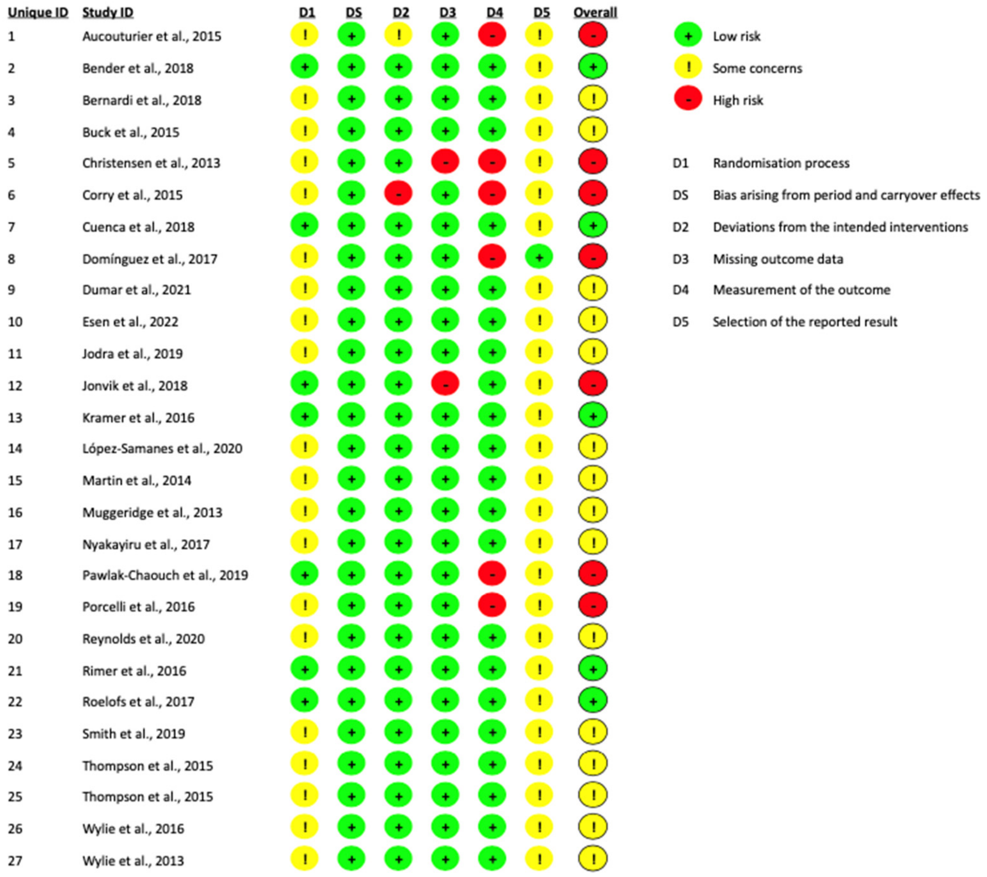

Figure S1. Risk of bias summary for individual for crossover trials [34–41,51–67,74,75]

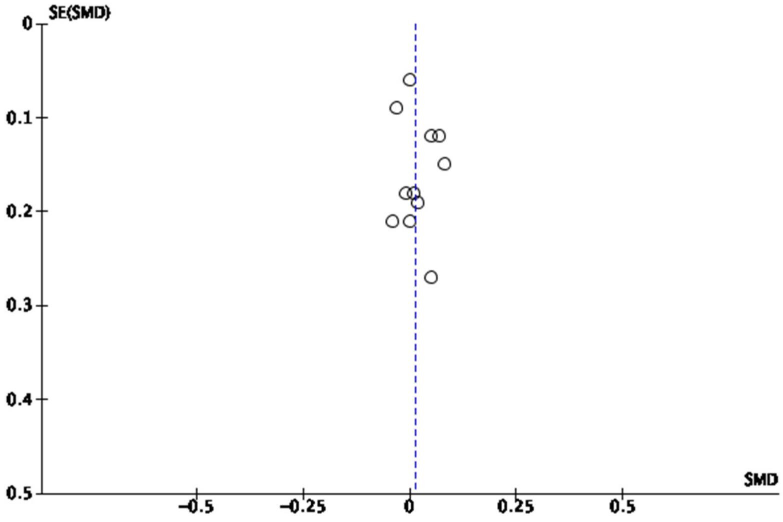

Figure S2. Funnel plot evaluating publication bias of trials assessing mean peak power output (n = 12).

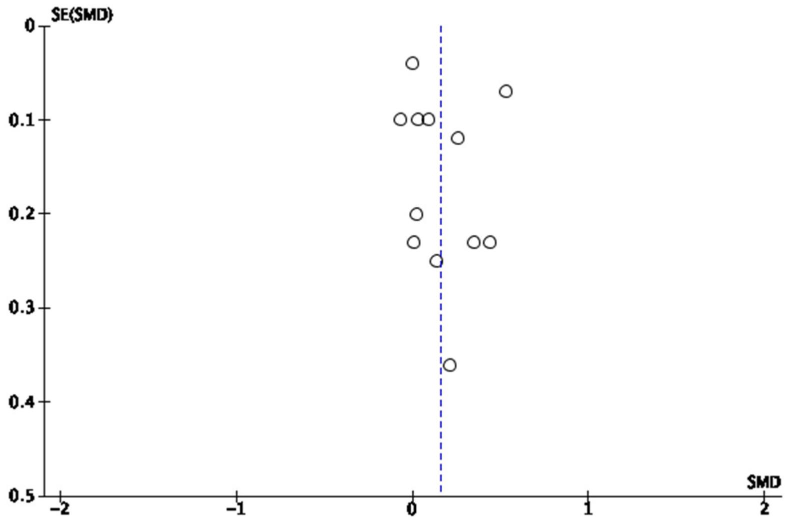

Figure S3. Funnel plot evaluating publication bias of trials assessing mean of the mean power output (n =12).

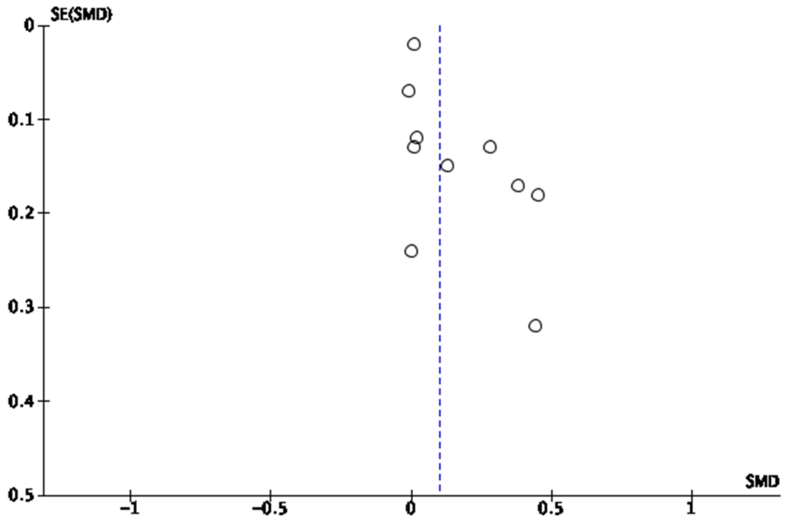

Figure S4. Funnel plot evaluating publication bias of trials assessing mean power output during the first sprint (n =10).

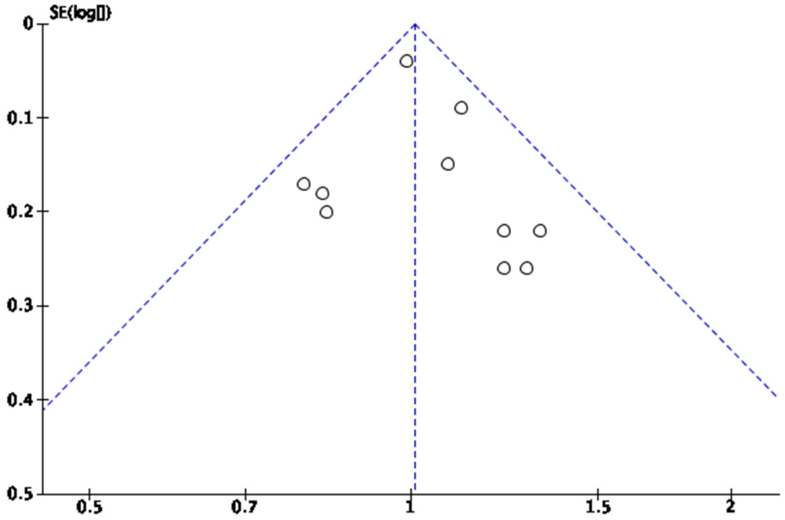

Figure S5. Funnel plot evaluating publication bias of trials assessing mean power output during the last sprint (n =10).

a)

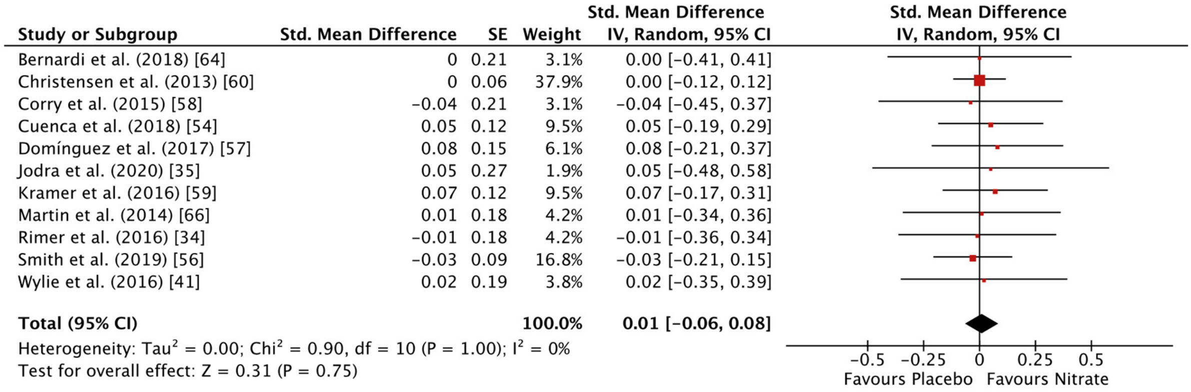

b)

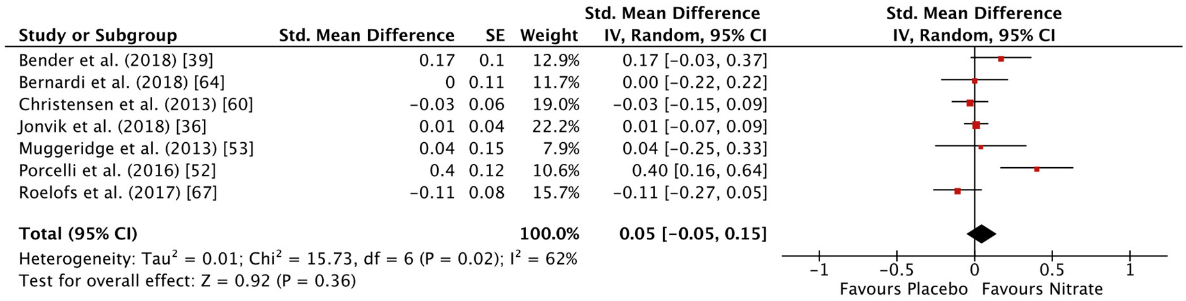

c)

Study or Subgroup	Std. Mean Difference	SE	Weight	Std. Mean Difference IV, Random, 95% CI
Bender et al. (2018) [39]	−0.34	0.1	14.2%	−0.34 [−0.54, −0.14]
Bernardi et al. (2018) [64]	0.31	0.11	13.7%	0.31 [0.09, 0.53]
Christensen et al. (2013) [60]	0.05	0.16	11.0%	0.05 [−0.26, 0.36]
Jonvik et al. (2018) [36]	0.03	0.04	17.0%	0.03 [−0.05, 0.11]
Muggeridge et al. (2013) [53]	0.17	0.07	15.8%	0.17 [0.03, 0.31]
Porcelli et al. (2016) [52]	0.58	0.12	13.1%	0.58 [0.34, 0.82]
Roelofs et al. (2017) [67]	−0.02	0.08	15.3%	−0.02 [−0.18, 0.14]
**Total (95% CI)**			**100.0%**	**0.10 [−0.06, 0.27]**

Heterogeneity: $Tau^2 = 0.04$; $Chi^2 = 43.93$, df = 6 ($P < 0.00001$); $I^2 = 86\%$
Test for overall effect: Z = 1.21 (P = 0.23)

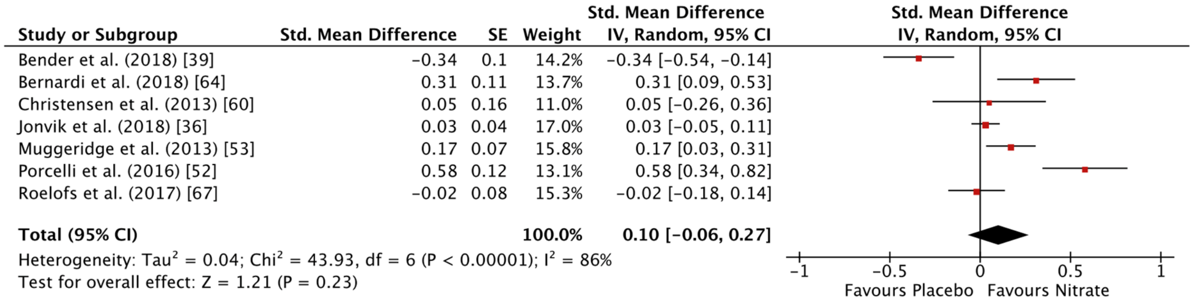

Figure S6: Forrest plot for mean peak power output (a), peak power during the first sprint (b), and peak power during the last sprint (c) in the nitrate and placebo trials.

a)

Study or Subgroup	Std. Mean Difference	SE	Weight	Std. Mean Difference IV, Random, 95% CI
**3.2.1 Low dose**				
Smith et al. (2019) [56]	−0.07	0.1	12.4%	−0.07 [−0.27, 0.13]
Martin et al. (2014) [66]	0.03	0.19	8.7%	0.03 [−0.34, 0.40]
Jodra et al. (2020) [35]	0.14	0.25	6.7%	0.14 [−0.35, 0.63]
Cuenca et al. (2018) [54]	0.26	0.12	11.6%	0.26 [0.02, 0.50]
Domínguez et al. (2017) [57]	0.35	0.23	7.3%	0.35 [−0.10, 0.80]
Dumar et al. (2021) [61]	0.67	0.24	7.0%	0.67 [0.20, 1.14]
**Subtotal (95% CI)**			**53.8%**	**0.19 [−0.02, 0.40]**
Heterogeneity: $Tau^2 = 0.04$; $Chi^2 = 11.43$, df = 5 (P = 0.04); $I^2 = 56\%$				
Test for overall effect: Z = 1.78 (P = 0.08)				
**3.2.2 High dose**				
Kramer et al. (2016) [59]	0.02	0.2	8.4%	0.02 [−0.37, 0.41]
Aucouturier et al. (2015) [65]	0.09	0.1	12.4%	0.09 [−0.11, 0.29]
Wylie et al. (2016) [41]	0.21	0.34	4.6%	0.21 [−0.46, 0.88]
Bernardi et al. (2018) [64]	0.44	0.23	7.3%	0.44 [−0.01, 0.89]
Corry et al. (2015) [58]	0.53	0.07	13.5%	0.53 [0.39, 0.67]
**Subtotal (95% CI)**			**46.2%**	**0.27 [0.01, 0.54]**
Heterogeneity: $Tau^2 = 0.06$; $Chi^2 = 16.39$, df = 4 (P = 0.003); $I^2 = 76\%$				
Test for overall effect: Z = 2.01 (P = 0.04)				
**Total (95% CI)**			**100.0%**	**0.23 [0.06, 0.40]**

Heterogeneity: $Tau^2 = 0.05$; $Chi^2 = 35.49$, df = 10 (P = 0.0001); $I^2 = 72\%$
Test for overall effect: Z = 2.65 (P = 0.008)
Test for subgroup differences: $Chi^2 = 0.23$, df = 1 (P = 0.63), $I^2 = 0\%$

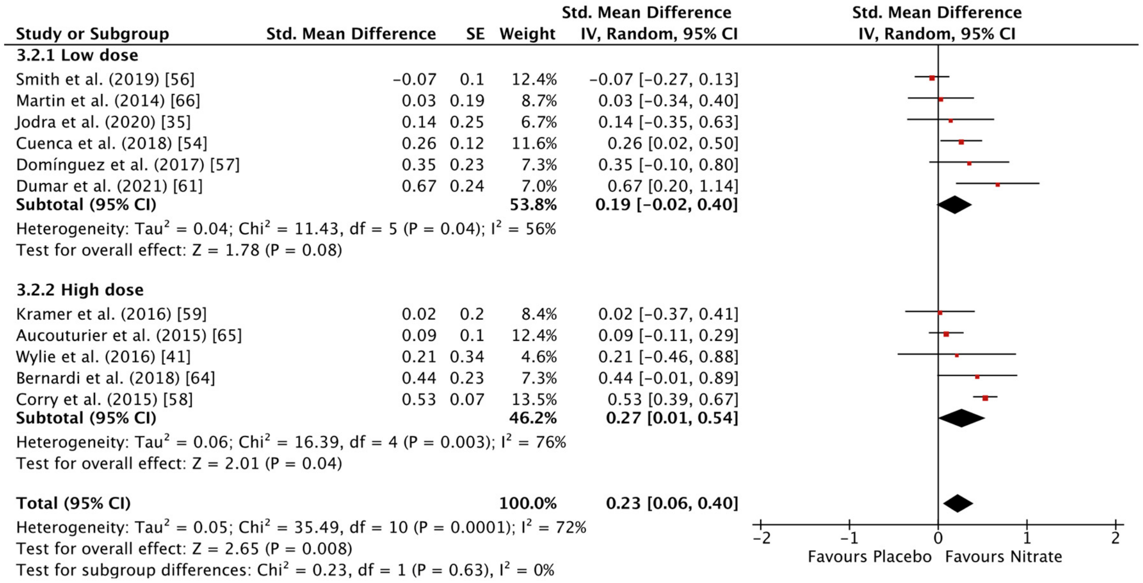

b)

Study or Subgroup	Std. Mean Difference	SE	Weight	Std. Mean Difference IV, Random, 95% CI
**4.2.1 Single day supplementation**				
Smith et al. (2019) [56]	−0.07	0.1	12.2%	−0.07 [−0.27, 0.13]
Martin et al. (2014) [66]	0.03	0.1	12.2%	0.03 [−0.17, 0.23]
Jodra et al. (2020) [35]	0.14	0.25	6.3%	0.14 [−0.35, 0.63]
Cuenca et al. (2018) [54]	0.26	0.12	11.3%	0.26 [0.02, 0.50]
Dumar et al. (2021) [61]	0.35	0.23	6.9%	0.35 [−0.10, 0.80]
Domínguez et al. (2017) [57]	0.35	0.23	6.9%	0.35 [−0.10, 0.80]
**Subtotal (95% CI)**			**55.7%**	**0.12 [−0.03, 0.26]**
Heterogeneity: $Tau^2 = 0.01$; $Chi^2 = 7.52$, df = 5 (P = 0.18); $I^2 = 34\%$				
Test for overall effect: Z = 1.59 (P = 0.11)				
**4.2.2 Multiple days supplementation**				
Kramer et al. (2016) [59]	0.02	0.2	7.9%	0.02 [−0.37, 0.41]
Aucouturier et al. (2015) [65]	0.09	0.1	12.2%	0.09 [−0.11, 0.29]
Wylie et al. (2016) [41]	0.21	0.36	3.9%	0.21 [−0.50, 0.92]
Bernardi et al. (2018) [64]	0.44	0.23	6.9%	0.44 [−0.01, 0.89]
Corry et al. (2015) [58]	0.53	0.07	13.4%	0.53 [0.39, 0.67]
**Subtotal (95% CI)**			**44.3%**	**0.27 [0.01, 0.54]**
Heterogeneity: $Tau^2 = 0.06$; $Chi^2 = 16.37$, df = 4 (P = 0.003); $I^2 = 76\%$				
Test for overall effect: Z = 2.00 (P = 0.05)				
**Total (95% CI)**			**100.0%**	**0.20 [0.04, 0.37]**
Heterogeneity: $Tau^2 = 0.05$; $Chi^2 = 36.00$, df = 10 (P < 0.0001); $I^2 = 72\%$				
Test for overall effect: Z = 2.46 (P = 0.01)				
Test for subgroup differences: $Chi^2 = 1.01$, df = 1 (P = 0.32), $I^2 = 0.9\%$				

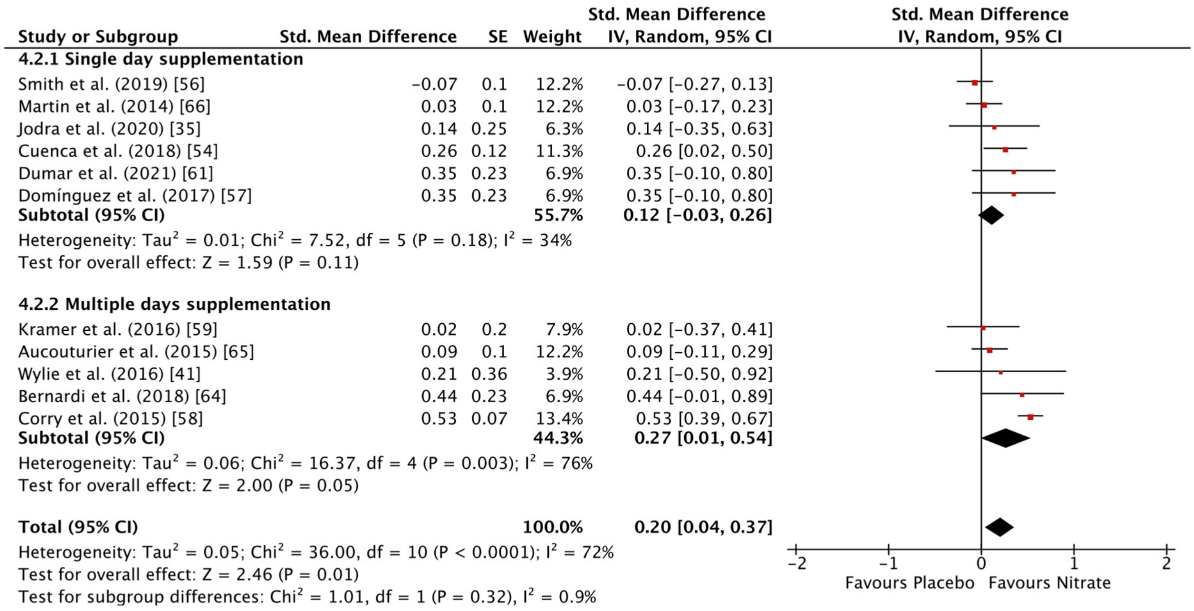

c)

Study or Subgroup	Std. Mean Difference	SE	Weight	Std. Mean Difference IV, Random, 95% CI
**5.2.1 Single Sprint**				
Kramer et al. (2016) [59]	0.02	0.2	8.1%	0.02 [−0.37, 0.41]
Jodra et al. (2020) [35]	0.14	0.25	6.5%	0.14 [−0.35, 0.63]
Cuenca et al. (2018) [54]	0.26	0.12	11.2%	0.26 [0.02, 0.50]
Domínguez et al. (2017) [57]	0.35	0.23	7.1%	0.35 [−0.10, 0.80]
Corry et al. (2015) [58]	0.53	0.07	13.1%	0.53 [0.39, 0.67]
**Subtotal (95% CI)**			**46.0%**	**0.31 [0.10, 0.51]**
Heterogeneity: $Tau^2 = 0.03$; $Chi^2 = 9.53$, df = 4 (P = 0.05); $I^2 = 58\%$				
Test for overall effect: Z = 2.92 (P = 0.004)				
**5.2.2 Repeated Sprints**				
Smith et al. (2019) [56]	−0.07	0.1	12.0%	−0.07 [−0.27, 0.13]
Martin et al. (2014) [66]	0.03	0.1	12.0%	0.03 [−0.17, 0.23]
Aucouturier et al. (2015) [65]	0.09	0.1	12.0%	0.09 [−0.11, 0.29]
Wylie et al. (2016) [41]	0.21	0.36	4.1%	0.21 [−0.50, 0.92]
Bernardi et al. (2018) [64]	0.44	0.23	7.1%	0.44 [−0.01, 0.89]
Dumar et al. (2021) [61]	0.67	0.24	6.8%	0.67 [0.20, 1.14]
**Subtotal (95% CI)**			**54.0%**	**0.14 [−0.04, 0.32]**
Heterogeneity: $Tau^2 = 0.02$; $Chi^2 = 11.13$, df = 5 (P = 0.05); $I^2 = 55\%$				
Test for overall effect: Z = 1.53 (P = 0.13)				
**Total (95% CI)**			**100.0%**	**0.23 [0.06, 0.39]**
Heterogeneity: $Tau^2 = 0.05$; $Chi^2 = 39.03$, df = 10 (P < 0.0001); $I^2 = 74\%$				
Test for overall effect: Z = 2.62 (P = 0.009)				
Test for subgroup differences: $Chi^2 = 1.44$, df = 1 (P = 0.23), $I^2 = 30.6\%$				

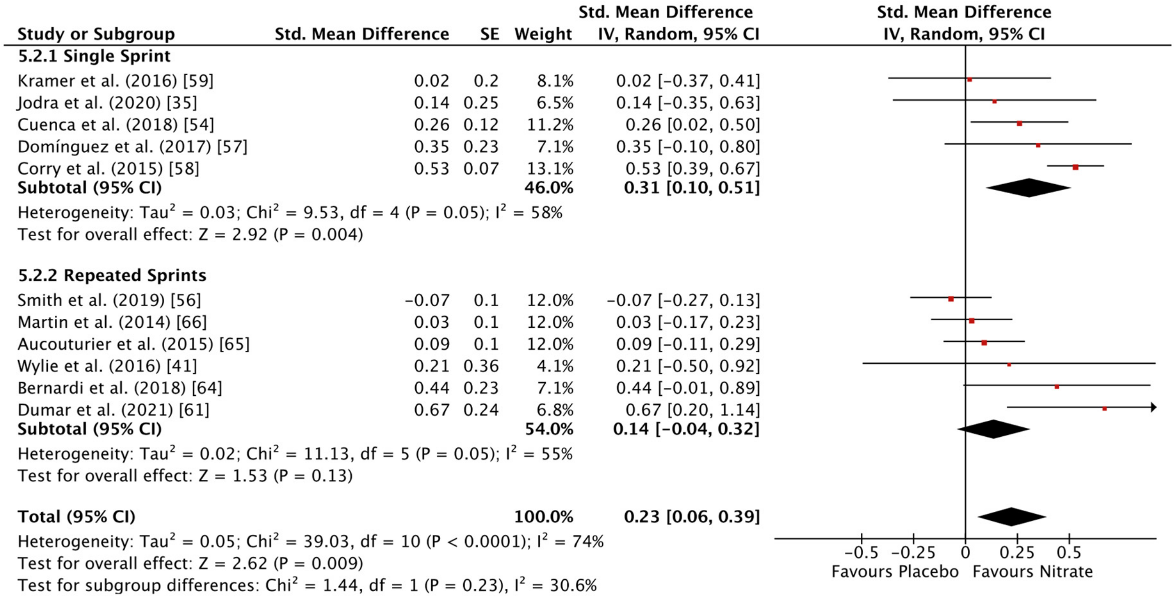

d)

Study or Subgroup	Std. Mean Difference	SE	Weight	Std. Mean Difference IV, Random, 95% CI
**6.2.1 Exercise duration ≤ 15 s**				
Smith et al. (2019) [56]	−0.07	0.1	12.0%	−0.07 [−0.27, 0.13]
Martin et al. (2014) [66]	0.03	0.1	12.0%	0.03 [−0.17, 0.23]
Aucouturier et al. (2015) [65]	0.09	0.1	12.0%	0.09 [−0.11, 0.29]
Bernardi et al. (2018) [64]	0.44	0.23	7.1%	0.44 [−0.01, 0.89]
Dumar et al. (2021) [61]	0.67	0.24	6.8%	0.67 [0.20, 1.14]
**Subtotal (95% CI)**			**49.9%**	**0.14 [−0.05, 0.34]**
Heterogeneity: $Tau^2 = 0.03$; $Chi^2 = 10.99$, df = 4 (P = 0.03); $I^2 = 64\%$				
Test for overall effect: Z = 1.44 (P = 0.15)				
**6.2.2 Exercise duration >15 s–≤ 30**				
Kramer et al. (2016) [59]	0.02	0.2	8.1%	0.02 [−0.37, 0.41]
Jodra et al. (2020) [35]	0.14	0.25	6.5%	0.14 [−0.35, 0.63]
Wylie et al. (2016) [41]	0.21	0.36	4.1%	0.21 [−0.50, 0.92]
Cuenca et al. (2018) [54]	0.26	0.12	11.2%	0.26 [0.02, 0.50]
Domínguez et al. (2017) [57]	0.35	0.23	7.1%	0.35 [−0.10, 0.80]
Corry et al. (2015) [58]	0.53	0.07	13.1%	0.53 [0.39, 0.67]
**Subtotal (95% CI)**			**50.1%**	**0.31 [0.12, 0.50]**
Heterogeneity: $Tau^2 = 0.02$; $Chi^2 = 9.83$, df = 5 (P = 0.08); $I^2 = 49\%$				
Test for overall effect: Z = 3.18 (P = 0.001)				
**Total (95% CI)**			**100.0%**	**0.23 [0.06, 0.39]**
Heterogeneity: $Tau^2 = 0.05$; $Chi^2 = 39.03$, df = 10 (P < 0.0001); $I^2 = 74\%$				
Test for overall effect: Z = 2.62 (P = 0.009)				
Test for subgroup differences: $Chi^2 = 1.42$, df = 1 (P = 0.23), $I^2 = 29.5\%$				

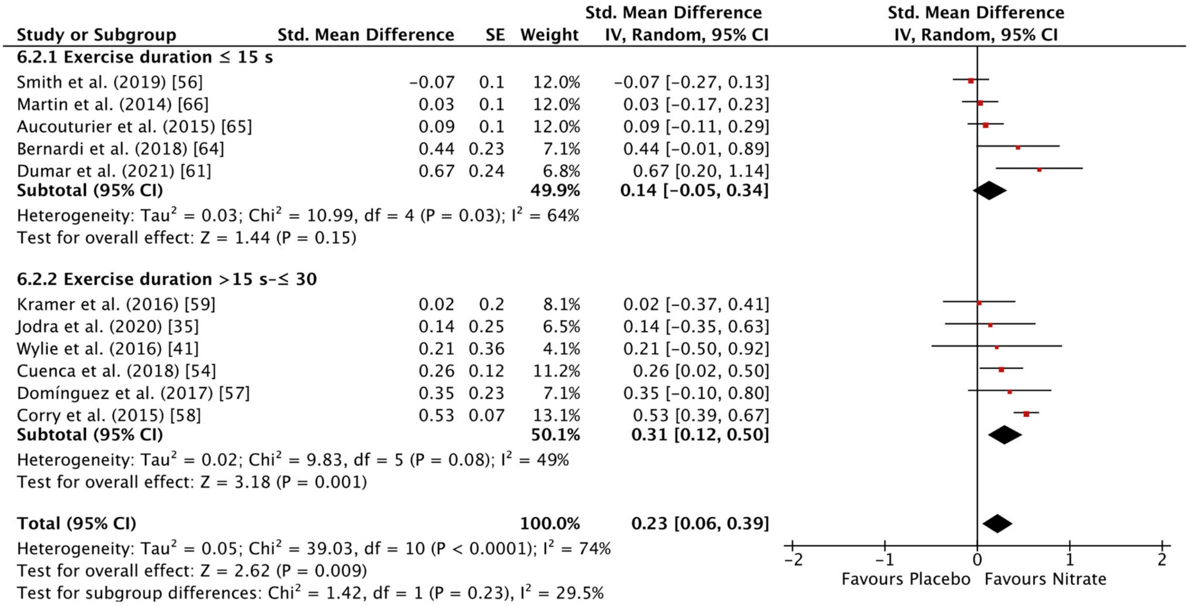

Figure S7. Forrest plot for mean power output sub-group analyse; low nitrate dose < 8 mmol compared to high nitrate dose ≥ 8 mmol (a), single day nitrate supplementation compared multiple days nitrate supplementation (b), single sprint compared to repeated sprints (c), exercise duration ≤ 15 s compared to exercise duration >15 s - ≤ 30 (d)

Study or Subgroup	Std. Mean Difference	SE	Weight	Std. Mean Difference IV, Random, 95% CI
Aucouturier et al. (2015) [65]	0.48	0.11	15.2%	0.48 [0.26, 0.70]
Dumar et al. (2021) [61]	0.25	0.32	6.5%	0.25 [−0.38, 0.88]
Martin et al. (2014) [66]	−0.31	0.1	15.7%	−0.31 [−0.51, −0.11]
Pawlak-Chaouch et al. (2019) [63]	0	0.12	14.7%	0.00 [−0.24, 0.24]
Rimer et al. (2016) [34]	0	0.1	15.7%	0.00 [−0.20, 0.20]
Smith et al. (2019) [56]	−0.07	0.09	16.1%	−0.07 [−0.25, 0.11]
Thompson et al. (2015) [51]	0.22	0.09	16.1%	0.22 [0.04, 0.40]
**Total (95% CI)**			**100.0%**	**0.06 [−0.13, 0.26]**
Heterogeneity: $Tau^2 = 0.06$; $Chi^2 = 34.40$, df = 6 (P < 0.00001); $I^2 = 83\%$				
Test for overall effect: Z = 0.64 (P = 0.52)				

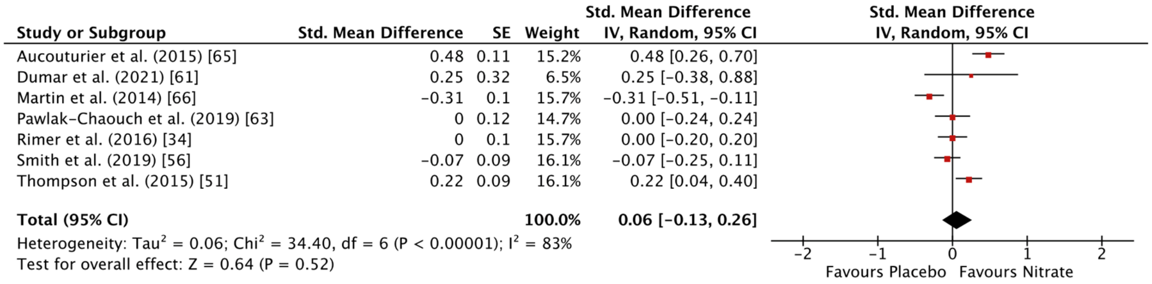

Figure S8: Forrest plot for total work done in the nitrate and placebo trials.
